# Novel domestic building energy consumption dataset: 1D timeseries and 2D Gramian Angular Fields representation

**DOI:** 10.1016/j.dib.2023.108985

**Published:** 2023-02-13

**Authors:** Abdullah Alsalemi, Abbes Amira, Hossein Malekmohamadi, Kegong Diao

**Affiliations:** a Institute of Artificial Intelligence, De Montfort University, Leicester, UK; b Department of Computer Science, University of Sharjah, Sharjah, UAE; c Institute of Energy and Sustainable Development, De Montfort University, Leicester, UK

**Keywords:** Energy efficiency, Internet of things, Environmental sensing, Occupancy, Smart plug, Image processing, Visualization

## Abstract

This data article describes a dataset collected in 2022 in a domestic household in the UK. The data provides appliance-level power consumption data and ambient environmental conditions as a timeseries and as a collection of 2D images created using Gramian Angular Fields (GAF). The importance of the dataset lies in (a) providing the research community with a dataset that combines appliance-level data coupled with important contextual information for the surrounding environment; (b) presents energy data summaries as 2D images to help obtain novel insights using data visualization and Machine Learning (ML). The methodology involves installing smart plugs to a number of domestic appliances, environmental and occupancy sensors, and connecting the plugs and the sensors to a High-Performance Edge Computing (HPEC) system to privately store, pre-process, and post-process data. The heterogenous data include several parameters, including power consumption (W), voltage (V), current (A), ambient indoor temperature (°C), relative indoor humidity (RH%), and occupancy (binary). The dataset also includes outdoor weather conditions based on data from The Norwegian Meteorological Institute (MET Norway) including temperature (°C), outdoor humidity (RH%), barometric pressure (hPA), wind bearing (deg), and windspeed (m/s). This dataset is valuable for energy efficiency researchers, electrical engineers, and computer scientists to develop, validate, and deploy and computer vision and data-driven energy efficiency systems.

**Specifications Table**

**Table:** 

Subject	Renewable Energy, Sustainability and the Environment
Specific subject area	Appliance-level electric power consumption with ambient environmental conditions in domestic building
Type of data	Timeseries Generated Images from Timeseries Figures Charts
How the data were acquired	The dataset was acquired using a smart plugs and ambient environmental sensors. The sensors were installed in a kitchen, living area, and a room of a household. The data was collected through a combination of local WiFi (smart plugs) and ZigBee protocol (environmental sensors). The data was collected on an edge computing hub, the ODROID-XU4 equipped with WiFi and CC2531 wireless dongles. It uses specialized energy management, Home Assistant, to store, pre- and post-process data. The following instruments were used in acquiring the data: 7 LocalBytes Power Monitoring Smart Plug for power consumption, 2 SONOFF SNZB-03 for occupancy detection, 3 SONOFF SNZB-02 for temperature and humidity measurement. Outdoor weather conditions were aggregated based on data from MET Norway.
Data format	Raw Analyzed
Description of data collection	The data was collected in a domestic household between April 2022 and November 2022 in a household in the United Kingdom from eleven sensors. Initially, the heterogenous data has been collected in time-series format. Additionally, the data has been later transformed into a two-dimensional (2D), heat map-like format using Gramian Angular Fields (GAF) for to aid in classification and data visualization. In the case of the 2D dataset, the readings were normalized using min-max normalization.
Data source location	*• City/Town/Region: Leicester* *• Country: United Kingdom*
Data accessibility	Repository name: Novel domestic building energy consumption dataset: 1D timeseries and 2D Gramian Angular Fields collection Data identification number: 10.17632/v2wr7grbbg.1 Direct URL to data: http://dx.doi.org/10.17632/v2wr7grbbg.1

## Value of the Data

• Data driven research: The data is valuable to the research community in which researchers can use to train Machine Learning (ML) energy data classification models on 1D timeseries and 2D GAF appliance-level data;
• Comprehensive parameters: The heterogenous dataset provides a large set of useful parameters that combine appliance-level data and contextual information for the surrounding environment such as temperature, humidity, and occupancy; and
• Visualization: The data also presents energy data summaries as 2D images to help obtain novel insights from using data visualization.

## 1 Objective

In the advancing field of energy efficiency, developing robust computational methods for analyzing energy efficiency behavior models necessitates creating correspondingly robust and rich dataset [1]. In domestic households, collecting appliance-level and ambient environmental data can lead to producing more effective energy efficiency models using Artificial Intelligence (AI) methods [2]. This is especially applicable when working on energy-saving research for domestic households where economic intrinsic incentives exist to save for the cost and the environment [3]. Accordingly, this dataset aims to help researchers train ML energy classification and recommender systems using 1D and 2D data formats, whereby, classical time-series models can be utilized as well as well-developed Deep Learning (DL) systems that can perceive the intricate details of GAF-generated snapshot of data.

## 2 Data Description

The dataset described is stored in three data containers (1) raw data for power consumption time-series, (2) raw data for ambient environmental conditions, and (3) raw 2D GAF data. The data format includes tables as Comma-Separated Values (CSV) files and images as Portable Network Graphics (PNG) files inside either folders or compressed zip files.

### 2.1 Raw Data: Power Consumption Time Series

A collection of tables (CSV files) that depict pre-processed power appliance-level consumption data as follows:

- Sheet 1: Power consumption of plug 1 (Television).
- Sheet 2: Power consumption of plug 2 (Kettle).
- Sheet 3: Power consumption of plug 3 (Computer Setup 1).
- Sheet 4: Power consumption of plug 4 (Toaster).
- Sheet 5: Power consumption of plug 5 (Washing Machine).
- Sheet 6: Power consumption of plug 6 (Computer Setup 2).
- Sheet 7: Power consumption of plug 7 (Fridge).

Each sheet includes the following columns: timestamp in*%year-%month-%day%hour:%minute:%second.% (*%Y-%m-%d%H:%M:%S.%f*)* format, parameter value (W), and UNIX timestamp.

### 2.2 Raw Data: Ambient Environment Time Series

A collection of tables (CSV files) that depict pre-processed ambient indoor (sheets 1–6) and outdoor (7) environment conditions as follows:

- Sheet 1: Temperature in kitchen/living room (°C).
- Sheet 2: Temperature in office room (°C).
- Sheet 3: Humidity in kitchen/living room (RH%).
- Sheet 4: Humidity in office room (RH%).
- Sheet 5: Occupancy in kitchen (binary).
- Sheet 6: Occupancy in living room (binary).
- Sheet 7: Outdoor weather data based on data from MET Norway temperature (°C), outdoor humidity (RH%), barometric pressure (hPA), wind bearing (deg), and windspeed (m/s).

Each sheet includes the following columns: timestamp in%Y-%m-%d%H:%M:%S.%f format, parameter value, and UNIX timestamp, with the exception of the outdoor weather data which is organized as follows: datetime%Y-%m-%d%H:%M:%S format UNIX timestamp, weather state, temperature, humidity, barometric pressure, wind bearing (direction), and wind speed.

### 2.3 Analyzed Data: 2D GAF Energy Data

Zip files representing data for the following:

- Power consumption of plug 1 (Television).
- Power consumption of plug 2 (Kettle).
- Power consumption of plug 3 (Computer Setup 1).
- Power consumption of plug 4 (Toaster).
- Power consumption of plug 5 (Washing Machine).
- Power consumption of plug 6 (Computer Setup 2).
- Temperature in kitchen/living room.
- Temperature in office room.
- Humidity in kitchen/living room.
- Humidity in office room.
- Occupancy in kitchen.
- Occupancy in living room.

In the above, each file includes:

- Files list (CSV): includes a description of all the training GAF files
- GAF raw data folder: includes raw GAF data, each image min-max normalized to 1-hour snapshots

**Fig. 1:**
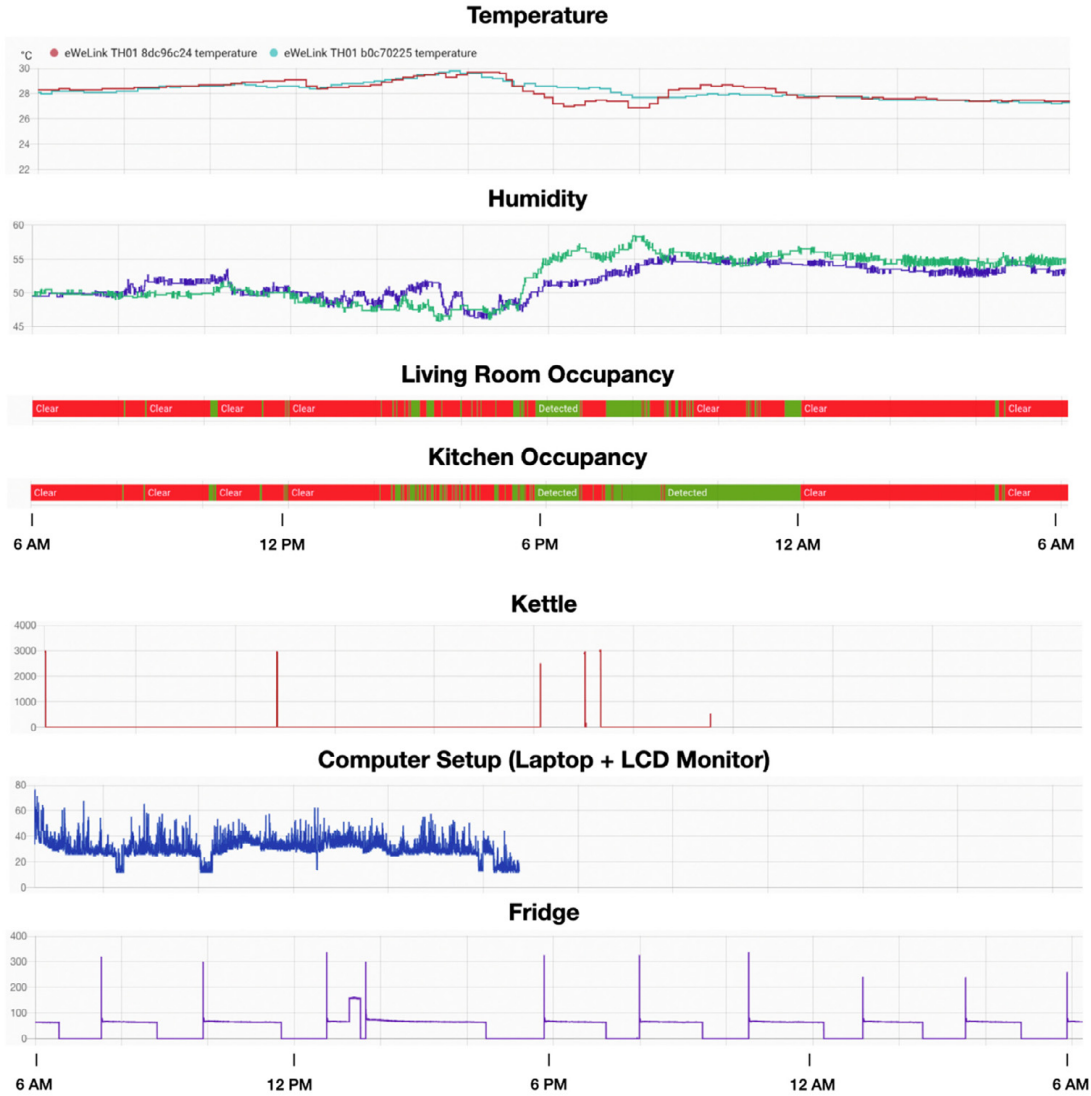
Examples of collected time-series data (from top to bottom) including temperature, humidity, occupancy, kettle, computer setup, and fridge.

**Fig. 2:**
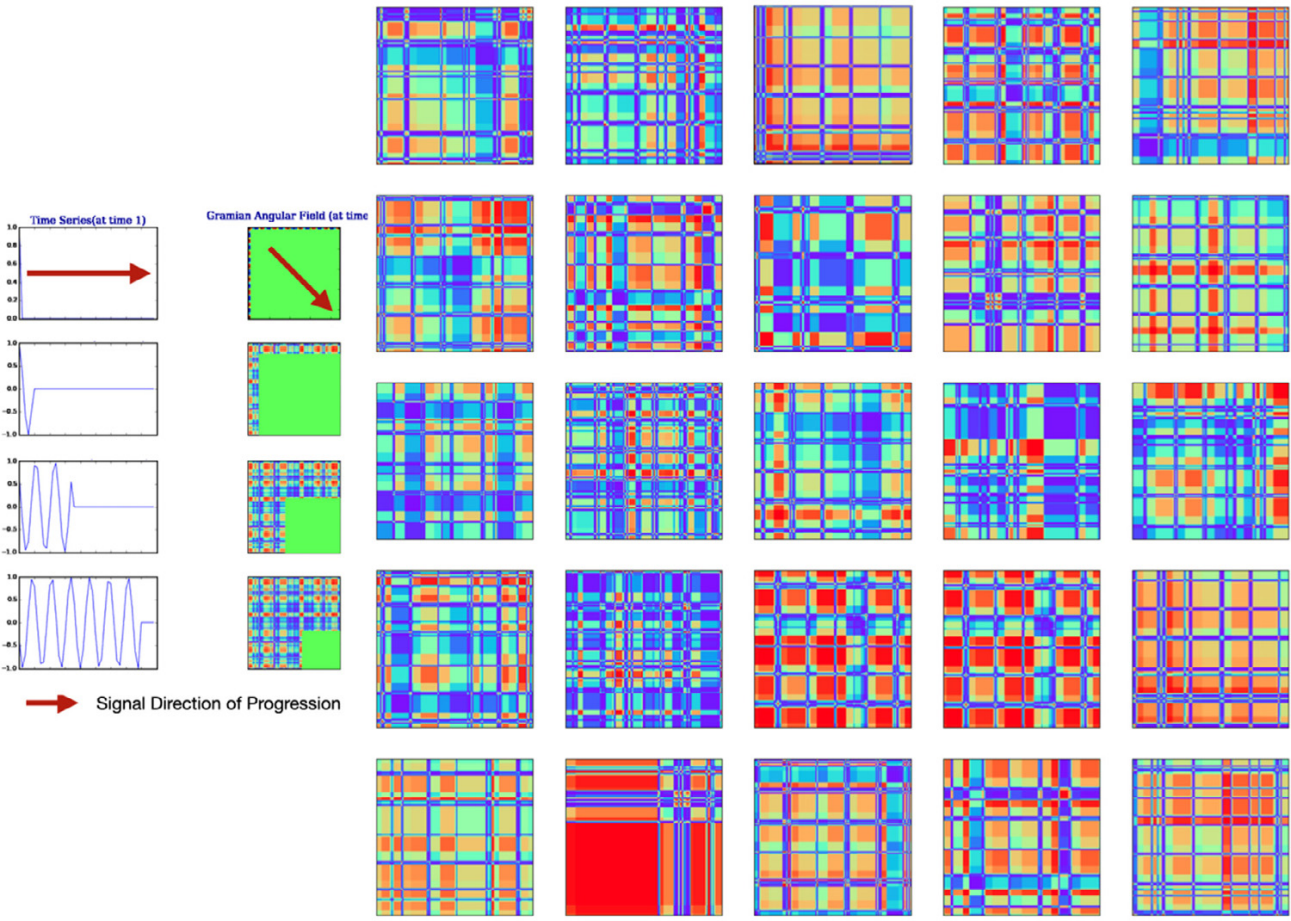
Sample 2D GAF images of 1-hour snapshots of energy consumption data of a TV.

Examples for time-series data are depicted in Fig. 1. Also, sample raw GAF data is shown in Fig. 2.

## 3 Experimental Design, Materials and Methods

**Fig. 3:**
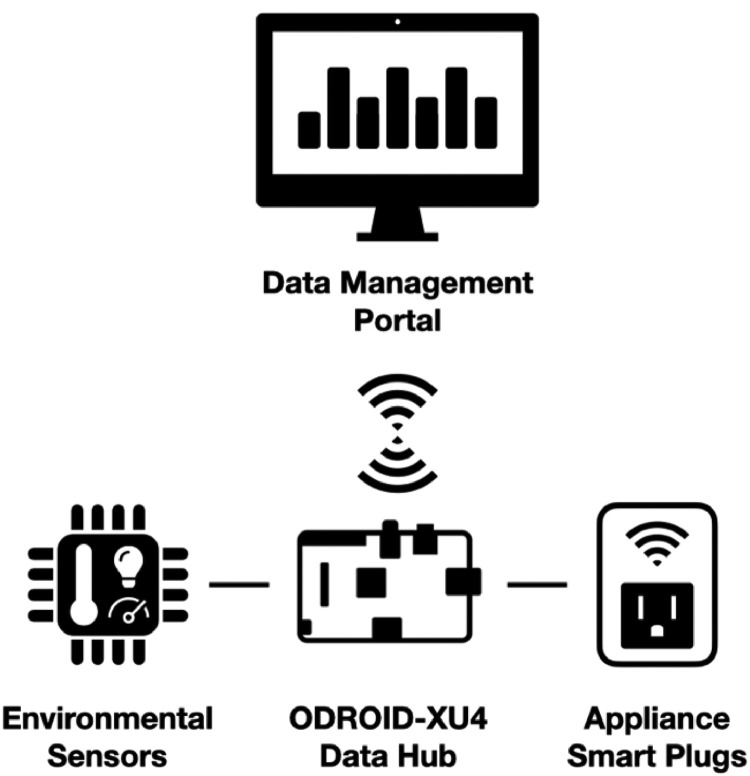
Overview of the Data Collection Setup. Edge computing board icon obtained from fredly from the Noun Project.

To start, the data is collected at a small-size residential household in the UK. Prior to configuring sensors and smart plugs, a central data management edge computing hub is needed. Fig. 3 shows the main data collection setup components. For this dataset, we have used the ODROID-XU4 edge platform, which has been chosen based on its cost and performance effectiveness in data-driven workflows [4]. Connected to a local network using an Ethernet cable, the ODROID-XU4 runs Home Assistant, which is an open-source smart home management system. Initially, the raw data is collected in the Structured Query Language (SQLite) format before further post-processing.

Afterwards, smart plugs and sensors are installed in specified locations at the household. First, smart plugs are connected to appliances including a kettle, TV, toaster, computer setup, fridge, and washing machine. Every plug is calibrated separately with a power meter to minimize reading errors. Also, environmental condition sensors, namely temperature, humidity, and occupancy sensors are placed in strategic locations (e.g., occupancy sensors are placed at the corner of a given room in order to maximize occupancy detection angle) to capture contextual information that support power consumption data. The occupancy sensors use Passive InfraRed (PIR) technology for presence detection. Similarly, the temperature and humidity sensors are calibrated against reference meters to ensure accuracy. The smart plugs and sensors are installed in the living room, kitchen, and study room. Table 1 describes the dataset's appliances.

**Table 1:** Specifications of appliances used in the dataset.

#	Appliance Name	Manufacturer	Power Rating (W)	Location
1	Television	Hisense	74	Living Room
2	Kettle	Tesco	2550–3000	Kitchen
3	Computer Setup 1 (MacBook Pro + Dell Monitor)	Apple/Dell	61/18	Office Room
4	Toaster	Tesco	750	Kitchen
5	Washing Machine	Indesit	2200	Kitchen
6	Computer Setup 2 (laptop + charging hub)	Hewlett Packard	119.9/38	Office Room
7	Fridge	Iceking	70–100	Kitchen

Once all smart plugs and sensors are installed and configured, they are tested and validated through wireless connectivity tests, occupancy detection accuracy tests, and data integrity tests on the ODROID-XU4. Table 2 shows parameter validation of the used sensors.

**Table 2:** Overview of sensor validation.

Parameter	Sensor	Average Accuracy
Temperature	SONOFF SNZB-02	±1 °C
Humidity	SONOFF SNZB-02	±5%
Occupancy	SONOFF SNZB-03	Up to 6 m (100°)
Power	LocalBytes Power Monitoring Smart Plug	2–3.5%

With data acquisition frequency averaging between 1 and 5 s, the dataset grows in size quite quickly. In the span of seven months, the data has reached more than 8.5 million datapoints. As continuous readings are accumulated into Home Assistant's SQLite database file, the data grows quite large, exceeding more than 5 GB. Accordingly, the raw database is restructured to only have the required data columns for further post-processing (i.e., timestamp, appliance name, power consumption, temperature, humidity, and occupancy). Following, the data is exported to a CSV file, after which the file size of the database is significantly compressed. It is noteworthy to mention that the outdoor condition's data acquisition frequency varies depending on the received data from MET Norway live data sources.

As described earlier, 1D time series are also transformed into a GAF representation by (a) converting cartesian points to the polar coordinates and using Gramian Angular Summation Field (GASF) [5,6] in Eq. (1) [7]:

(1) $$GASF=\left(\begin{array}{ccc} \cos\left(\emptyset_{1}+\emptyset_{1}\right) & \cdots & \cos\left(\emptyset_{1}+\emptyset_{N}\right)\\ \vdots & \ddots & \vdots \\ \cos\left(\emptyset_{N}+\emptyset_{1}\right) & \cdots & \cos\left(\emptyset_{N}+\emptyset_{N}\right) \end{array}\right)$$

where ∅ the is derived in Eq. (2):

(2) $$\{\emptyset =\arccos\left({\tilde{x}}_{l}\right),\;0<{\tilde{x}}_{l}<1\in x$$

Henceforth, the timeseries data is fed into the GAF processing program developed in [8] to produce 2D GAF image files, each file representing a 1-hour fragment of the data.

## Ethics Statements

This data was collected in accordance the Declaration of Helsinki and have obtained ethical approval from Faculty of Computing, Engineering and Media at De Montfort University (CEM ID No G414200).

## CRediT authorship contribution statement

**Abdullah Alsalemi:** Conceptualization, Methodology, Writing – original draft, Writing – review & editing, Validation. **Abbes Amira:** Conceptualization, Methodology, Writing – review & editing, Validation, Supervision. **Hossein Malekmohamadi:** Conceptualization, Methodology, Writing – review & editing, Validation, Supervision. **Kegong Diao:** Conceptualization, Methodology, Writing – review & editing, Validation, Supervision.

## Declaration of Competing Interest

The authors declare that they have no known competing financial interests or personal relationships that could have appeared to influence the work reported in this paper.

## Data Availability

Novel domestic building energy consumption dataset: 1D timeseries and 2D Gramian Angular Fields representation (Original data) (Mendeley Data)
